# 

**Published:** 1884-12

**Authors:** 


HALL'S
### JOURNAL OF HEALTH
—AND—
### MEDICINE.
MONTHLY.
E. H. GIBBS, A. M., M. D., EDITOR.
FOUNDED IN 1854.

### EDITORIAL ANNOUNCEMENT.

THIRTY-ONE years ago, Dr. W. W. Hall, a remarkably successful and widely-known physician of New York, established this publication, to be, as its name implies, a "Journal of Health."

By this he meant, not only a medical publication, containing items of interest to the physician, but a magazine devoted to the wants of the people, and a text-book to be used in the family.

It was not contemplated to issue a series of essays on *Physiology* or *Hygiene*, nor enter into technical disquisitions on the chemical analysis of food, the philosophy of cell life and development; nor indeed any systematic expositions, but simply in short articles, in plain English, to treat of such subjects as bear on these great points:—How to determine disease in its first approach; how to arrest it at once by natural agencies; how to live to prevent sickness. The world would hail it as a glad event, if physicians could be so educated as to cure all diseases, but it would more largely add to its happiness if all could be so well instructed as to the first symptoms of every ailment, as to be able to arrest at once its progress. *To this end do we labor.*

*To teach men how to avoid disease* was the idea which first prompted the determination to publish this periodical, and everything that has been published in it during the past thirty.one years has been designed to tend more or less in that direction.

The success which attended the appearance of this magazine, and the reputation which it soon won, of being the *first popular* health publication in the world, shows that its founder not only understood the wants of the people but was fully able to supply them.

For several years before the founder's death the present editor was his colleague in the editorship of the JOURNAL, and he believes that time has fully shown that he has continued to carry out successfully the ideas advanced above.

In this, then, the closing number of the year, we solicit the renewal of subscriptions, believing that the coming year will be a new era in the life of the JOURNAL. Some changes are in progress which will make its appearance more attractive, and hereafter it will be issued promptly on the 20th of the month preceding that of the date on title page.

The subscription price will remain at $1.00 a year.

